# Expert views on state-level naloxone access laws: a qualitative analysis of an online modified-Delphi process

**DOI:** 10.1186/s12954-022-00645-1

**Published:** 2022-06-08

**Authors:** Sean Grant, Rosanna Smart

**Affiliations:** 1 Department of Social and Behavioral Sciences, Indiana University Richard M. Fairbanks School of Public Health, 1050 Wishard Blvd, RG 6046, Indianapolis, IN 46202 USA; 2 Economics, Sociology, and Statistics Department, RAND Corporation, 1776 Main Street, Santa Monica, CA 90401 USA

**Keywords:** Delphi, Naloxone, Opioids, Overdose, Pharmacy, Policy

## Abstract

**Background:**

Expanding availability to naloxone is a core harm reduction strategy in efforts to address the opioid epidemic. In the US, state-level legislation is a prominent mechanism to expand naloxone availability through various venues, such as community pharmacies. This qualitative study aimed to identify and summarize the views of experts on state-level naloxone access laws.

**Methods:**

We conducted a three-round modified-Delphi process using the online ExpertLens platform. Participants included 46 key stakeholders representing various groups (advocates, healthcare providers, human/social service practitioners, policymakers, and researchers) with expertise naloxone access laws. Participants commented on the effectiveness and implementability of 15 state-level naloxone access laws (NALs). We thematically analyzed participant comments to summarize views on NALs overall and specific types of NAL.

**Results:**

Participants commented that the effectiveness of NALs in reducing opioid-related mortality depends on their ability to make sustained, significant impacts on population-level naloxone availability. Participants generally believed that increased naloxone availability does not have appreciable negative impacts on the prevalence of opioid misuse, opioid use disorder (OUD), and non-fatal opioid overdoses. Implementation barriers include stigma among the general public, affordability of naloxone, and reliance on an inequitable healthcare system.

**Conclusions:**

Experts believe NALs that significantly increase naloxone access are associated with less overdose mortality without risking substantial unintended public health outcomes. To maximize impacts, high-value NALs should explicitly counter existing healthcare system inequities, address stigmatization of opioid use and naloxone, maintain reasonable prices for purchasing naloxone, and target settings beyond community pharmacies to distribute naloxone.

## Introduction

Mortality from opioid-related overdoses remains a significant public health issue [1], with a reversal in recent progress during the COVID-19 pandemic [2, 3]. Consequently, expanding naloxone availability remains a core harm reduction strategy to address the opioid epidemic in the US [4, 5]. State-level naloxone access laws (NALs) aim to stimulate greater naloxone availability to communities through various mechanisms, such as community pharmacies [6], community-based programs [7], emergency departments [8], and law enforcement initiatives [9]. Substantial evidence exists supporting efforts to reduce opioid-related overdose mortality through naloxone distribution [10].

Despite the priority of addressing the opioid crisis and empirical evidence supporting naloxone’s efficacy in reversing overdoses, there is substantial variation in state adoption of NALs [11], mixed results for the effectiveness of different NAL components [12–16], and growing partisanship regarding how best to address the crisis [17]. In the absence of clear and direct empirical evidence, formal consensus methods can help clarify what experts believe is (not) effective and implementable [18, 19]. Previous work found expert consensus on the average effectiveness and implementability of various state-level NALs [20], but there are numerous contextual considerations that may mediate or moderate the effects of policies as implemented in practice. Furthermore, given variability in potential barriers to effective and equitable implementation of NALs [21–25], it is important to clarify whether and why experts believe various policy options are (not) implementable. The objective of this qualitative study is summarizing experts’ rationale for ratings in an online-modified Delphi process on the extent to which NALs are effective, acceptable, feasible, affordable, and equitable.

## Material and methods

The study was deemed exempt by the RAND Human Subjects Protection Committee (ID-2018–0506). We prospectively registered the study on the Open Science Framework (https://osf.io/f4hk8/). A separate manuscript reports quantitative findings from the online modified-Delphi process [20]. We developed this manuscript using the Standards for Reporting Qualitative Research [26].

### Qualitative approach and researcher reflexivity

Our study was guided by the GRADE Evidence-to-Decision (EtD) Framework, which facilitates structured and transparent use of evidence to inform public health recommendations and decisions [27]. Our qualitative approach involved a post-positivist research paradigm employing a combination of coding grounded in the data and thematic analysis informed by constructs in the GRADE EtD Framework. The study authors have training in social science disciplines that emphasize evidence-informed decision-making and generally privilege quantitative methods for causal inference.

### Sampling strategy

We recruited participants identifying with one of several stakeholder groups (advocates, healthcare providers, human or social service practitioners, policymakers, and researchers). We first developed a recruitment list from published research related to NALs, project advisory board suggestions, and relevant organizations’ member lists. We also used a “snowball sampling” approach allowing approached stakeholders to nominate further participants. We then emailed a recruitment survey to potential participants to indicate whether they would be willing to participate in the online modified-Delphi process, offering a $300 gift card or prepaid debit card for study completion. Individuals indicating interest provided demographic data and stated their preference to participate in the panel focused on either (a) effectiveness or (b) implementability of NALs. Participants electronically provided informed consent, which included information about sharing de-identified information and assurances of protecting confidentiality of responses and discussion comments. We aimed to recruit 40–80 participants (20–40 participants per panel), based on guidance for online expert panels that aim to engage large, diverse, and geographically-distributed groups of stakeholders in consensus exploration [28].

### Data collection

We conducted two online modified-Delphi panels concurrently during summer 2020 using RAND’s ExpertLens system: one on NAL effectiveness and another on their implementability. We explicitly instructed participants that, while there are several critically important channels for naloxone distribution, we specifically were interested in understanding policies that target naloxone access and distribution through pharmacies (e.g., chain pharmacies, independent community pharmacies). In Round One, participants rated each policy on four outcomes (Effectiveness Panel) or implementability criteria (Implementation Panel). In Round Two, participants explored areas of agreement and disagreement by discussing Round One results in an anonymous, asynchronous online forum. In Round Three, participants re-rated Round One results following Round Two discussion [29]. Each round takes participants approximately one hour to complete and remains open for one to three weeks.

We constructed a list of 15 NALs through an iterative process of feedback from the project’s advisory board and cognitive testing on the ExpertLens platform (see Table 1). We developed criteria for assessing each policy using the GRADE EtD Framework [27] and APEASE framework from the Behaviour Change Wheel [30]. These frameworks underlie prominent approaches to evidence-informed decision-making via explicit criteria on the effectiveness, acceptability, feasibility, affordability and equitability of interventions under consideration (see Table 2).

**Table 1:** State-level naloxone access laws

Category	Policy	Definition
Liability policies	Liability protections for prescribers	Provide legal protections for healthcare professionals who prescribe naloxone in accordance with state law. Protections can extend to criminal liability; civil liability; and administrative, licensing, and professional disciplinary action by the prescriber’s professional licensure (or similar) entity
	Liability protections for dispensers	Provides liability protections for pharmacists who dispense naloxone in accordance with state law. Protections can extend to criminal liability; civil liability; and administrative, licensing, and disciplinary action by the state board of pharmacy (or similar entity)
	Liability protections for administration of naloxone	Provide liability protections to laypersons or nonmedical professionals (e.g., law enforcement officers) who administer naloxone. Protections can extend to criminal liability; civil liability; and professional sanctions
Education/training requirements	Prescriber provision of education or training to naloxone recipients	Requires prescribers of naloxone to offer overdose training and/or education to the recipient of a naloxone prescription
	Dispenser provision of education or training to naloxone recipient	Requires pharmacists to offer overdose training and/or education to the recipient of a naloxone prescription
Co-prescribing naloxone	Co-prescribing laws based on opioid dosage only	Require doctors to prescribe naloxone to patients taking high doses of opioid painkillers
	Co-prescribing laws based on more than opioid dosage	Require doctors to prescribe naloxone to patients who have other risk indicators for opioid overdose above and beyond taking high doses of opioid painkillers (e.g., patients in opioid treatment programs, patients with a prior history of opioid use disorder or overdose)
Layperson accessibility	Third party prescription	Allows a healthcare provider with naloxone-prescribing authority to prescribe to an at-risk person’s family member, friend, and/or other person in a position to assist the at-risk person in the event of an opioid-related overdose
	Over-the-counter pharmacy supply	Makes naloxone available as an ordinary retail purchase that does not require a prescription. For this policy, assume that the US Food and Drug Administration has changed the prescribing status of naloxone from prescription‐only to over‐the‐counter status
Expanded pharmacy access	Population-based collaborative agreement	Pharmacists are given permission to voluntarily enter into collaborative agreements (or standing orders) with physicians and other providers to dispense naloxone to eligible patients without a patient-specific prescription according to patient criteria and instructions defined by the authorizing prescriber
	Statewide standing or protocol order	Establish a statewide framework that allows any pharmacist in the state (who meets qualifications specified in the protocol) to dispense naloxone without a patient-specific prescription under the pre-defined conditions outlined in the order. Unlike collaborative practice agreements, this policy does not require pharmacists to have a partnering prescriber
	Pharmacist prescriptive authority	Involves the legislature expanding pharmacist scope of practice to allow pharmacists to directly prescribe or furnish naloxone to patients without any physician involvement
Cost subsidization	Insurance coverage	Requires health insurance plans to provide coverage for at least one generic opioid antagonist and device approved to treat opioid overdose (e.g., naloxone) without prior authorization
	State subsidies for naloxone purchase through insurance	Involves states providing co-pay assistance to individuals purchasing naloxone through health insurance plans that include prescription coverage, including Medicaid and Medicare as well as commercial insurance
	Statewide “free naloxone”	Allows any resident to visit any pharmacy across the state and anonymously obtain naloxone at no cost without an individual prescription or appointment

**Table 2:** Criteria for assessing state-level naloxone access laws

Domain	Criterion	Definition
Effectiveness	Naloxone pharmacy distribution	Amount of naloxone dispensed through retail pharmacies (e.g., chain pharmacy stores, independent community pharmacies)
	Opioid use disorder prevalence	Percentage of the general population with a pattern of opioid use leading to clinically and functionally significant impairment, health problems, or failure to meet major responsibilities
	Nonfatal opioid overdose	Per capita rates of nonfatal overdose related to opioids, including opioid analgesics (e.g., oxycodone), illegal opioids (e.g., heroin), and synthetic opioids (e.g., fentanyl)
	Opioid overdose mortality	Per capita rates of fatal opioid overdose
Implementability	Acceptability	The extent to which the policy is acceptable to the general public in the state or community where the policy has been enacted
	Feasibility	The extent to which it is feasible for a state or community that has enacted the policy to implement it as intended
	Affordability	The extent to which the resources (costs) required to implement the policy are affordable from a societal perspective
	Equity	The extent to which the policy is equitable in its impact on health outcomes across populations of people who use opioids

### Data analysis

To examine stakeholders’ views on NAL effectiveness and implementability, we systematically coded all comments from the two rating rounds and discussion round [31, 32]. The first author (SG) initially grouped all comments by NAL and criterion, ordered comment groupings by stakeholders’ numeric ratings, and thoroughly read and re-read the material [33, 34]. SG then conducted line-by-line coding, relating the raw data to codes that were grounded in the data itself and that emerged through constant comparison and refinement during coding. Lastly, SG systematically indexed the codes into preliminary themes, revised and integrated similar themes, relabeled the final themes, and identified quotations best exemplifying themes. The second author (RS) reviewed all codes, themes, and supporting quotations.

## Results

Of 94 potential participants, 46 (48.9%) agreed to participate: 24 in the panel on NAL effectiveness (Panel A) and 22 in the panel on NAL implementability (Panel B). Most panelists were female (54%), White Non-Hispanic (78%), identified as researchers (61%), and resided in the Northeast (41%). We did not identify any discernable differences in final themes by participant demographics. Overall, participants provided 2,658 comments: 1,479 in Round One (698 in Panel A, 781 in Panel B), 319 in Round 2 (123 in Panel A, 196 in Panel B), and 860 in Round Three (478 in Panel A, 382 in Panel B). Across all comments, participants cited several studies as warrant for their claims [6, 12, 15, 35–41].

### Overall themes

We organized overall themes by the domains of the GRADE EtD Framework: effectiveness, acceptability, feasibility, affordability, and equity (see Table 3).

**Table 3:** Overall themes for specific categories of the evidence-to-decision framework

Categories	Themes	Exemplary quotes
Effectiveness	Pharmacies are limited as a setting for naloxone distribution	“Pharmacies themselves will tend to be a suboptimal vehicle for getting naloxone to people most likely to experience or witness an overdose” (Participant B03)
	NALs that make it easy and affordable for anyone to obtain naloxone without a prescription have more substantial impacts on pharmacy naloxone distribution	“When naloxone is in the hands of people who use drugs and their communities, and is accessible free and in a low-barrier way that can eliminate stigma, hassle, insurance concerns, people will access it” (Participant A08)
	NALs that do not increase naloxone distribution substantially will not reduce opioid-related mortality	“I think that the increase in distribution is likely small and thus these second order effects are likely to be even smaller” (Participant A11)
	NALs do not directly impact OUD prevalence or nonfatal opioid overdoses	“I am not sure OUD prevalence would be affected anyway by any of these laws and provisions” (Participant A26)
	NALs may indirectly have small and acute impacts on OUD prevalence and nonfatal opioid overdoses	“More naloxone→fewer opioid deaths→increased OUD prevalence through less loss of people, but will NOT cause new OUD” (Participant A06)
		“Largely mechanical: increased provision of naloxone→reduction in fatal opioid overdose mortality→increase in non-fatal opioid overdose mortality” (Participant A17)
Acceptability	“High acceptability” as evidence that states have implemented specific NALs with little blowback	“Given how many states have done this with little blowback, it seems quite acceptable to the public” (Participant B15)
	“High acceptability” as a positive trend in recent years of public support for naloxone access	“Naloxone prescribing and distribution faced a lot of opposition before being more commonly endorsed by public agencies in the past decade” (Participant B03)
	“High acceptability” as a lack of opposition due to a lack of public awareness of the existence of NALs	“I think the general public would largely be unaware of such a law” (Participant B11)
	“Moderate acceptability" due to remaining stigma around naloxone and substance use	“Public still hates people who use drugs. Many want to punish them, not treat them” (Participant B14)
		“Risk compensation, where the general public thinks giving out naloxone prescriptions encourages drug use, could reduce general public acceptability” (Participant B24)
Feasibility	NAL feasibility depends on levels of buy-in from stakeholders involved in implementation	“Assuming that the stakeholders agreed on this policy, it should be relatively simple to implement” (Participant B04)
	NAL feasibility depends on existing resources and infrastructure in relevant settings	“The infrastructure is already in place to make this happen” (Participant B07)
	“Moderate feasibility" often due to remaining stigma around naloxone and substance use	“There is a "not in my lobby" mentality… toward people who use drugs. Some [providers] think that if they do not offer MAT, naloxone… they will deter patients who use drugs from their facility/site. These stigmas may mean despite the policy, pharmacies refuse to participate in practice” (Participant B24)
Affordability	Naloxone costs significantly impact NAL affordability	“The "policy" and the cost of the "naloxone" are two different things. The naloxone [itself] can be pricy” (Participant B09)
	Naloxone costs vary due to numerous factors (e.g., market forces on naloxone pricing, type of naloxone product)	“Without insurance, the cost of intranasal Narcan … is cost prohibitive. In addition, many pharmacies do not carry the cheaper, generic injectable naloxone” (Participant B13)
	Who pays for naloxone significantly impacts NAL affordability	“May cost the state/community money to pay for the naloxone” (Participant B22)
	The cost-effectiveness of NALs with significant reductions in mortality improves their affordability	“Cost-effective due to reduced morbidity and mortality related to overdoses, first responders, and emergency room care” (Participant B06)
Equity	Systemic discrimination and structural oppression counter potential equitability of NALs	“Mandates that do not consider racial or other socioeconomic factors are anticipated to be equitable. However … the law itself is equitable, but subject to the foundational inequities of our society and healthcare system” (Participant B26)
	Interpersonal bias and discrimination counter potential equitability of NALs	“Individual biases would continue to impact patient identification and delivery of naloxone” (Participant B26)
	Pharmacies are often less accessible in rural areas and to subpopulations of people who use opioids	“That seems about as easy access as possible unless you live somewhere with no pharmacies within a reasonable distance and/or a person didn’t have transportation or access to transportation to actually get to a pharmacy” (Participant 17)
	Equitability is inversely related to out-of-pocket costs for naloxone	“This policy will improve equity by reducing cost barriers to prescribed naloxone” (Participant B18)

#### Effectiveness

Participants believe that numerous NALs can improve naloxone pharmacy distribution—and that several of these NALs can produce population-level reductions in opioid overdose mortality via greater probabilities of naloxone being present and administered during an overdose. However, participants consistently noted that meaningful reductions in mortality would be modest for any NAL that has pharmacy distribution as its mechanism of action, given pharmacies’ limitations as a setting for distributing naloxone. For example, many participants commented that barriers for pharmacy distribution include stigma towards people who use drugs, potential high out-of-pocket costs of naloxone, and community-based programs being more likely to reach those with higher risks of overdosing from opioids. Consequently, participants believed only NALs that make it easy and affordable for anyone to obtain naloxone without a prescription—and that align the stocking and dispensing of naloxone with routine pharmacy operations—have the potential for the substantial and sustainable increases in pharmacy naloxone distribution required for meaningful reductions in population-level overdose mortality.

Participants believed that NALs without links to prevention, screening, treatment, or recovery do not directly influence the prevalence of OUD or nonfatal opioid overdoses, as NALs primarily target opioid-related overdose mortality. That said, NALs that substantially increase naloxone distribution may indirectly have small and acute impacts on these outcomes: i.e., large reductions in fatal opioid overdoses result in more individuals with OUD surviving non-fatal overdoses. Four participants explicitly expressed concerns about this dynamic creating a moral hazard of naloxone distribution causing purposeful opioid misuse: i.e., concerns that people who use drugs will engage in riskier drug behaviors if they have access to naloxone. However, the consensus view was that NALs do not lead to “risk compensation”, but rather any increase in population-level OUD or non-fatal overdoses is a mechanical consequence of reducing fatal overdoses.

#### Acceptability

Participants rating NALs as highly acceptable to the general public often commented about actual implementation of specific NALs with little pushback, positive trends of public support for naloxone access, or lack of opposition due to lack of awareness of NAL existence. Nonetheless, participants also consistently noted that acceptability is still mired by stigma about people who use drugs—and naloxone as encouraging opioid use by extension. Participants offered strategies for mitigating this stigma, namely accompanying the passage of NALs with effective framing of naloxone as an evidence-based response to the opioid epidemic, and messaging that NALs would not increase opioid misuse.

#### Feasibility

Participants saw the feasibility of implementing NALs as dependent on existing resources and infrastructure, as well as levels of buy-in from stakeholders involved in implementation. Existing resources and infrastructure that modify feasibility included sustainable funding, health information technology, and physical space. Participants also noted that feasibility depends on whether an NAL adds new roles and responsibilities for the physicians, other prescribers, and pharmacists involved in naloxone distribution, as these professionals already feel overburdened by their current workloads: “requiring additional pharmacist time at the point of dispensing may be difficult” (B05). As with acceptability, participants noted stigma substantially influences stakeholder support for NALs and consequently NAL feasibility across a range of organizations and settings. Specific stakeholders included first responders, healthcare organizations, healthcare and social service providers, insurance companies, law enforcement, lawyers and legal experts, licensing boards, naloxone manufacturers, pharmacists and pharmacy chains, prescribers, professional associations, regulators, and state legislatures. Organizations and settings included including clinics, healthcare systems, hospitals, and pharmacies.

#### Affordability

Variability in ratings on the societal affordability of NALs was largely a factor of the cost of naloxone itself, who pays for naloxone, and effectiveness of an NAL in reducing overdoses. The cost of naloxone significantly influenced views on affordability because naloxone is the most significant direct and ongoing cost of NALs, and the cost of naloxone can vary due to numerous factors (e.g., impact of NAL on naloxone market pricing, type of naloxone product covered under an NAL). Consequently, as part of their rationale for affordability ratings, participants frequently commented on naloxone-related cost burdens for specific stakeholders (e.g., insurance coverage, out-of-pocket costs, pharmacy stocking costs, state purchasing, and subsidization costs). From a societal perspective, many participants rating NALs as highly affordable noted cost savings associated with decreased overdose mortality.

#### Equitability

Variability in equitability ratings largely related to inequities and disparities of society and the systems in which NALs are implemented, rather than the equitability of NALs per se. Namely, participants frequently commented on structural, systemic, and institutional oppression many populations face in the USA due to their race, ethnicity, socioeconomic status, and place of residence—and how the resultant unjust stratification of health opportunities and outcomes are likely to replicate in implementation of NALs that rely on pharmacies and the existing healthcare system. In addition, participants often identified discrimination and interpersonal biases held by healthcare providers and pharmacists as another potential source of inequitable implementation. Participants also noted pharmacies are a suboptimal mechanism for distributing naloxone to people most likely to experience or witness an overdose, as pharmacies are often less prevalent in specific areas (e.g., small, rural) and less accessible to specific populations (e.g., low-income). Consequently, even NALs that increase the number of places and ways that people can access naloxone still could widen disparities in naloxone access if implemented solely through pharmacies. Lastly, participants generally posited that lowering out-of-pocket naloxone cost increases an NAL’s equitability, particularly for low-income and under- or uninsured populations.

### Themes for specific NALs

We organized themes for specific NALs by policy groupings: liability policies, education and training requirements, co-prescribing naloxone, layperson accessibility, expanded pharmacy access, and cost subsidization (see Table 4).

**Table 4:** Themes for Specific Categories of NAL

Categories	Themes	Exemplary quotes
Liability policies	Criminal, civil, and administrative liability are not major concerns of prescribers and dispensers	“Liability concern is not a major hindrance to prescribing/distributing naloxone” (Participant A03)
	In-principle support of liability protections for nonmedical administrators of naloxone, though no meaningful impact on pharmacy naloxone distribution	“The people most at risk for naloxone related liability are other people who use drugs. I don’t think that many get naloxone from pharmacies (but I could be wrong). I don’t think this policy change would increase pharmacy based naloxone distribution very much” (Participant A12)
	Broad public acceptability of protecting providers and laypersons addressing the opioid epidemic	“Highly acceptable to the public that an individual not be punished for doing what they could to assist another in good faith” (Participant B26)
	Feasible and affordable due to lack of implementation challenges and costs once passed	“This is a legal protection and does not require significant labor for implementation” (Participant B18)
	“Moderate equitability" because these laws do not address existing disparities of access to and biases in the healthcare system	“Would not address biases in healthcare against people with mental health issues, who experience homelessness, or who live in poverty” (Participant B04)
Education/training requirements	Onerous nature of these requirements would lead to less prescribing and dispensing of naloxone	“If burdensome training prevents prescribing of naloxone, then benefits of education/training efficacy for those with naloxone may be offset by lower naloxone access” (Participant A15)
	Acceptability of receiving information about proper usage for other medications extends to naloxone	“Consultations and education on proper usage is afforded for all other prescriptions, it should be here as well” (Participant B07)
	Implementability concerns related to time, reimbursement, training of trainers, and infrastructure needed to provide confidential patient education	“It’s entirely possible to offer training in flexible ways that don’t require prescribers to deliver the training (videos, websites, handouts, etc.)…"feasibility" really turns in great part on WHO is to do the training and WHAT modality is required” (Participant B09)
	Equitability concerns due to disproportionate negative impact of burdens from this mandate on marginalized and underserved communities	“Could be an equalizer because education is required, but if it results in providers being more selective about who they prescribe to … then it could create inequalities given some of the research about race/ethnic differences in opioid prescribing and access to MOUD” (Participant B11)
Co-prescribing naloxone	Strong evidence that these policies expand access to naloxone through pharmacies	“I still believe the data that when higher-risk people get co-prescribed, the greatest number of naloxone will go out” (Participant A20)
	Only modest decreases in mortality due to focus on populations who are prescribed opioids rather than diverted prescriptions and illicit opioids	“Although it would decrease the mortality rate, most of the OD are not from prescription opioids, they are from illicit opioids (fentanyl)” (Participant A01)
	Negative reactions from patients being labelled as persons needing naloxone and providers being told what medications to prescribe and when	“Factoring pushback from providers who don’t want to be mandated to do things and from patients … who do not want to be "stigmatized" as having OUD” (Participant B05)
	Concerns about the feasibility and cost of these mandates	“The U.S. still prescribes more opioids than any nation on earth, even a 25% rate of co-prescription is going to cost a lot of money” (Participant B15)
	Relies on access to healthcare system for an issue (chronic pain) with documented racial and ethnic treatment disparities	“I see no reason it would address intersectional issues of equity among people of color, low income people, etc. who use drugs, especially considering people of color are less likely to be prescribed opioids” (Participant B24)
	Supportive in-principle of using risk indicators beyond opioid overdose, but concerned about actual implementability in-practice	“The law may not be as concrete and well defined when determining the other factors that are considered high risk for overdose and these more squishy factors could be differentially applied across demographic groups and thus worsen health disparities for certain populations” (Participant B11)
Layperson accessibility	Greater accessibility of naloxone to anyone (regardless of opioid use status) removes barriers to naloxone pharmacy distribution	“The more people that have access to a naloxone prescription, the more people there are getting it from the pharmacy” (Participant A26)
	Third party accessibility less effective on overdose mortality than OTC pharmacy supply due to reliance on physician prescriptions and targeting of laypersons not likely to be present during overdose	“For this intervention [third party accessibility] to decrease fatal OD … family and friends need to be near the individual who is injecting or using heroin or fentanyl (the most common causes of fatal OD). I’m concerned that … family might not be present when individual is using drugs” (Participant A01)
	Equitability concerns about prohibitory retail costs of OTC naloxone for low-income persons	“It [OTC] makes it easier to access, but it doesn’t make it affordable for those who are most vulnerable” (Participant B07)
Expanded pharmacy access	Facilitates significant naloxone pharmacy distribution by removing the need for physician involvement	“Putting the authority to prescribe Naloxone in the hands of the pharmacist and removing the additional barrier of having to go through a doctor would increase pharmacy distribution” (Participant A26)
	Publicly acceptable given several examples of successful adoption without much pushback	“This is happening all over without much pushback and other respondents seemed to share my assessment that the public is fine with this” (Participant B15)
	Feasible assuming pharmacist willingness and lack of opposition from prescribers	“Fairly straightforward, though depends on pharmacist willingness” (Participant B04)
	Affordable due to eliminating the costs associated with office visits with prescribers	“Reducing costs associated with seeking naloxone because it would not require an office visit, and instead someone could go to a pharmacy in the community whenever it is open” (Participant B24)
	Increased equitability from removing the need to access prescribers, but remaining concerns about pharmacist bias and limited access to pharmacies	“Can reach people who do not have access or relationship with a prescriber. Can improve equitable access to naloxone through available community pharmacies. Gaps would be in places without pharmacies” (Participant B18)
Cost subsidization	Significantly facilitate naloxone pharmacy distribution by addressing out-of-pocket costs	“Cost is often an issue for patients, so breaking down this barrier would improve access for patients” (Participant A14)
	Statewide free naloxone is the most equitably effective NAL but also the least acceptable to the public and affordable to the state	“Of all policies considered, this should necessarily have the largest effects. It both eliminates costs for patients and removes all supply-side barriers. Stigma will continue to put downward pressure on provision, but such a policy might even help reduce stigma over the longer term” (Participant A17)
	Insurance coverage is less equitable and effective but more implementable than statewide free naloxone due to burdens falling on insurance companies	“General public sentiment is that more medications should be covered” (Participant B01)
	State subsidies are less effective than statewide free naloxone and less implementable than insurance coverage because it only provides assistance with co-pays and costs fall on the state	“Opposition may come from those who wish to avoid spending taxpayer funds on PWUD, those who resent that insurance companies don’t pay the whole thing, and those who think limited funding should be directed elsewhere” (Participant B05)

#### Liability policies

Participants generally agreed that liability policies are ineffective because liability concerns are not a major barrier for naloxone prescribing and dispensing—even if liability protections may make some prescribers and pharmacists more comfortable prescribing and dispensing naloxone. Similarly, participants indicated in-principle support of liability protections for administration of naloxone by laypersons and first responders, although they did not view such policies as having a meaningful impact on naloxone distribution through pharmacies. The consensus that liability policies are acceptable to the public stemmed from perceptions of broad support for efforts to protect healthcare providers and laypersons acting in good faith to address the opioid epidemic. That said, several respondents noted that some members of the general public may be weary of the potential for liability protections to provide leeway for professional maleficence among prescribers and dispensers. Participants considered these policies as feasible and affordable primarily due to a lack of implementation challenges and ongoing costs once passed, especially because liability protection laws regarding naloxone are already well-established. Any concerns were related to potential pushback on the scope of liability protections during the legislative phase from law enforcement, licensing boards, professional societies, and trial lawyers. In contrast, participants generally considered liability policies only moderately equitable because these NALs at best do not counter and at worst risk replicating existing disparities of access to and biases in the healthcare system.

#### Education/training requirements

While considered acceptable to the general public, requirements of prescribers or pharmacists to offer training and education to recipients of naloxone were viewed as both ineffective and relatively less implementable. Participants postulated that these requirements create barriers to naloxone pharmacy distribution by increasing burdens on prescribers and dispensers, who are already pressed for time. The additional burden of this unfunded mandate would deter many physicians from prescribing and pharmacists from dispensing naloxone, offsetting any potential benefits of the education and training on knowledge about overdoses and competencies in using naloxone correctly.

Implementability concerns related to time constraints, reimbursement for training and education, the need to “train the trainers”, and the lack of infrastructure at pharmacies for confidential patient education. To mitigate these implementability concerns, numerous participants suggested offering education and training in flexible approaches and via streamlined technology (e.g., free, readily-available videos and handouts). For example, one participant noted that all communications and education tools from the federal Rural Health Opioid Program (Office of Rural Health Policy) are freely available for replication. Putting aside concerns about stigma of substance use and naloxone generally, perceived acceptability of these laws specifically stems from agreement among the general public to provide information about proper use for all medications. However, concerns about equitability arose by the interaction of the onerous aspects of this mandate with the structural oppression and interpersonal discrimination faced by many people and communities affected by these policies. Consequently, panel consensus reflected views that these policies likely disproportionately negatively impact marginalized communities, namely people of color, people with mental health disorders, people who are homeless, rural residents, and individuals with lower socioeconomic status or communities with less resources. That said, conditional on receiving training, these policies could improve equity among those who previously have been underserved in terms of medical education.

#### Co-prescribing naloxone

Naloxone co-prescribing requirements were generally viewed as effective in substantially increasing naloxone pharmacy distribution, but only modestly effective in reducing overdose mortality. While participants noted evidence that these policies expand access to naloxone through pharmacies [35–37], this substantially expanded access may not translate into large decreases in mortality because these policies focus on prescribed opioids rather than diverted prescriptions and illicit opioids. In addition, consensus ratings indicated only moderate implementability of these policies due to negative reactions from patients being labelled as persons needing naloxone, and providers being told what medications to prescribe and when to prescribe them. In addition, participants indicated concerns about ensuring that prescribers follow the mandate (e.g., through regulation, enforcement, and oversight). Another concern was the cost of this policy, given the number of opioids still prescribed in the USA and the perceived possibility for these policies to further incentivize pharmaceutical manufacturers to inflate naloxone prices. At the societal level, co-prescribing may be cost-effective through averted fatal opioid overdoses [42, 43], particularly if increased market competition from new naloxone products maintains affordability of naloxone itself [44]. Participants also raised concerns about the equitability of these policies, as they not only rely on access to pharmacies but also to access to prescribers—and for an issue (chronic pain) with documented racial and ethnic treatment disparities due to systemic racism and interpersonal biases [45].

Participants often showed support for the principle of using risk indicators beyond opioid overdose in order to cover overdose risk more broadly. However, participants noted numerous issues of this policy in-practice: patient perceptions of additional questions as invasive, provider burdens due to increased complexity and difficulty in obtaining this information (e.g., data privacy protections), uncertainty about the indicators to use (and subsequent waste in resources if the wrong indicators are used), and greater subjectivity (compared to the more objective criteria of using only prescribed opioid dosages) increasing the opportunity for interpersonal biases to yield further inequities.

#### Layperson accessibility

Participants generally viewed NALs facilitating greater accessibility of naloxone to anyone (regardless of opioid use status) as effective in removing barriers for laypersons and thereby increasing naloxone pharmacy distribution. Namely, over-the-counter (OTC) pharmacy supply was one of the NALs that participants believed had the best chance to destigmatize acquiring naloxone, because many third parties (especially family and caregivers) are likely to acquire naloxone via a pharmacy (rather than through a community-based naloxone distribution program). These ratings translated into perceptions of meaningful reductions in fatal overdoses from authorizing OTC naloxone pharmacy supply, but not from third party prescription due to the latter’s reliance on physician prescriptions and narrower focus on laypersons who may not actually witness overdoses. Conversely, participants viewed third party prescriptions as slightly more equitable than OTC pharmacy supply due to concerns about prohibitory retail costs of OTC naloxone for low-income persons. While both OTC supply and third-party prescribing had consensus ratings of “high” affordability, participants raised financial concerns for both (payer costs for third party prescribing, and patient out-of-pocket costs for OTC pharmacy supply), but viewed both as generally acceptable to the public and feasible changes to prescriber and dispenser practices.

#### Expanded pharmacy access

Participants also viewed expanded pharmacy access laws as effective in facilitating naloxone availability by removing patient barriers to acquiring naloxone (i.e., the need for patient-specific prescriptions and physician involvement) and enabling pharmacist autonomy. As evidence for their acceptability, participants frequently cited successful adoption of these NALs without much public pushback (likely due to their commensurability with obtaining other types of medication from pharmacists). Participants explained the “high feasibility” consensus was conditional on pharmacist willingness to dispense naloxone without physician involvement and lack of opposition from prescribers concerned about professional scope creep. “High affordability” was generally due to eliminating costs associated with prescriber office visits. Statewide standing/protocol orders and pharmacist prescriptive authority had the added benefit of lesser administrative costs compared to collaborative practice agreements (i.e., one statewide policy versus multiple agreements). Removing the need to access prescribers increased participant views of equitability, though participants noted the remaining possibilities of pharmacist bias and limited access to pharmacies.

#### Cost subsidization

Participants viewed cost-subsidization NALs as effective in significantly facilitating naloxone pharmacy distribution because they address the significant barrier of out-of-pocket costs to individuals. Participants particularly viewed statewide free naloxone as the most equitable NAL in effectively facilitating naloxone pharmacy distribution and reducing overdose mortality, given its elimination of out-of-pocket costs and potential for destigmatizing naloxone. However, they also considered it the least affordable policy for states—and least acceptable as a result. While insurance coverage was not seen as equitable and effective in reducing overdose mortality due to many high-risk patients not having insurance, participants viewed insurance coverage as the most implementable cost-subsidization policy due to public support of shifting burdens to insurance companies covering the medication. Participants similarly viewed state subsidies for naloxone purchase through insurance as less effective due to its reliance on high-risk patients having insurance, although they also saw it as less implementable than insurance coverage as it only provides assistance with co-pays (rather than full coverage) and costs fall on the state (rather than insurance companies).

## Discussion

Experts believed that the effectiveness of NALs in reducing opioid-related mortality requires sustained, significant impacts on population-level naloxone availability. This necessitates addressing implementation barriers that apply broadly across NALs, including affordability of naloxone itself, reliance on an inequitable healthcare system, and stigma—which can mitigate the effectiveness of all types of policies considered [46, 47]. Experts also generally believed that increased naloxone availability does not have appreciable negative impacts on the prevalence of opioid misuse, OUD, and non-fatal opioid overdoses. This contrasts with recent work suggesting increasing rates of both nonfatal opioid-related overdoses and opioid-related crime following standing order or third-party prescribing laws [48], but aligns with several studies showing no evidence that take-home naloxone provision promotes increased opioid use or overdose [49]. Additionally, while experts expect any short-term mechanical increases in OUD or non-fatal overdoses to be small, many do not think they are negligible or insignificant (i.e., policymakers should consider and plan for them).

Regarding specific NALs, expanded pharmacy access laws appeared to be the most valuable set of policies—with statewide standing or protocol orders considered particularly “high-value”—as these policies remove the most barriers to naloxone distribution and access. This does not imply these policies are absent implementation barriers, such as failure of pharmacies to stock naloxone and high out-of-pocket costs to purchasers [50]; thus, these policies may operate best when coupled with cost subsidization policies, although these are often expensive for states to implement. In contrast, laws requiring education or training to naloxone recipients were seen as "low-value" policies, both ineffective in improving health-related outcomes and burdensome to implement; this may be increasingly true in contexts where user-friendly naloxone formulations or naloxone training in non-medical settings are readily available [51]. Liability protections were supported but not seen as an effective mechanism for substantial naloxone pharmacy distribution, while co-prescribing laws involve a trade-off between widening eligibility criteria and provoking stigma around questions beyond opioid dosage. Lastly, prescriptions themselves are a barrier to layperson accessibility, as participants believed OTC naloxone provided a better option than third party prescriptions, particularly if accompanied by state subsidies or requirements that insurers cover OTC naloxone costs [52].

The following limitations should be considered when interpreting the study’s results. Our method required participants to have stable Internet access, proficiency with online survey systems, and several hours of availability over the three rounds. While our sample size is commensurate with recommendations for online modified-Delphi processes [28], we did not use random sampling procedures for our stakeholder populations. In addition, our recruited sample is entirely US-based, largely non-Hispanic white, and predominantly consisted of researchers. Lastly, each stakeholder could only participate in one panel (effectiveness or implementability) to reduce burden and attrition across rounds, which may have yielded different comments than stakeholders responding to both effectiveness and implementability criteria.

## Conclusions

Experts believe NALs that significantly increase naloxone access are associated with less overdose mortality without risking substantial unintended public health outcomes. To maximize impact, “high-value policies” explicitly counter existing inequities in the healthcare system, address stigmatization of opioid use and naloxone, maintain reasonable prices for purchasing naloxone, and target settings beyond community pharmacies to distribute naloxone.

## Data Availability

The prospectively registered protocol, pre-analysis plan, and study materials are on Open Science Framework (https://osf.io/6swq2).
